# Pharmacokinetic-Pharmacodynamic Modeling of the D_2_ and 5-HT_2A_ Receptor Occupancy of Risperidone and Paliperidone in Rats

**DOI:** 10.1007/s11095-012-0722-8

**Published:** 2012-03-22

**Authors:** Magdalena Kozielska, Martin Johnson, Venkatesh Pilla Reddy, An Vermeulen, Cheryl Li, Sarah Grimwood, Rik de Greef, Geny M. M. Groothuis, Meindert Danhof, Johannes H. Proost

**Affiliations:** 1 Division of Pharmacokinetics, Toxicology and Targeting, University of Groningen, P.O. Box 196, 9700 AD Groningen, The Netherlands; 2 Advanced PK-PD Modeling & Simulation, Janssen Research and Development Janssen Pharmaceutica NV, Beerse, Belgium; 3 Worldwide Research & Development, Pfizer, Inc., Groton, Connecticut USA; 4 Clinical PK-PD, Pharmacokinetics, Pharmacodynamics & Drug Metabolism, Merck Sharp & Dohme, Oss, The Netherlands; 5 Division of Pharmacology, Leiden/Amsterdam Center for Drug Research, Leiden, The Netherlands

**Keywords:** dopamine D_2_ receptor occupancy, mechanism-based PK-PD, paliperidone, risperidone, serotonin 5-HT_2A_ receptor occupancy

## Abstract

**Purpose:**

A pharmacokinetic-pharmacodynamic (PK-PD) model was developed to describe the time course of brain concentration and dopamine D_2_ and serotonin 5-HT_2A_ receptor occupancy (RO) of the atypical antipsychotic drugs risperidone and paliperidone in rats.

**Methods:**

A population approach was utilized to describe the PK-PD of risperidone and paliperidone using plasma and brain concentrations and D_2_ and 5-HT_2A_ RO data. A previously published physiology- and mechanism-based (PBPKPD) model describing brain concentrations and D_2_ receptor binding in the striatum was expanded to include metabolite kinetics, active efflux from brain, and binding to 5-HT_2A_ receptors in the frontal cortex.

**Results:**

A two-compartment model best fit to the plasma PK profile of risperidone and paliperidone. The expanded PBPKPD model described brain concentrations and D_2_ and 5-HT_2A_ RO well. Inclusion of binding to 5-HT_2A_ receptors was necessary to describe observed brain-to-plasma ratios accurately. Simulations showed that receptor affinity strongly influences brain-to-plasma ratio pattern.

**Conclusion:**

Binding to both D_2_ and 5-HT_2A_ receptors influences brain distribution of risperidone and paliperidone. This may stem from their high affinity for D_2_ and 5-HT_2A_ receptors. Receptor affinities and brain-to-plasma ratios may need to be considered before choosing the best PK-PD model for centrally active drugs.

## INTRODUCTION

Schizophrenia is a chronic psychiatric disorder which affects almost 1% of the population worldwide (1). It is characterized by the presence of positive symptoms (e.g. hallucinations, delusions), negative symptoms (e.g. social withdrawal, reduced motivation) and cognitive impairments. The majority of drugs for schizophrenia target psychotic symptoms as their primary goal (1). It has been hypothesized that elevated dopamine levels in the striatum lead to psychosis. This is consistent with the fact that all currently available antipsychotic drugs act as dopamine D_2_ receptor antagonists (with one exception of a partial agonist - aripiprazole) (1). Usually 65–80% D_2_ receptor occupancy (RO) is believed to be necessary for a clinically relevant outcome, but occupancy above 80% leads to side effects, i.e. Extra Pyramidal Symptoms (EPS)(2).

In addition to blocking D_2_ receptors, newer antipsychotics (so-called second generation or atypical antipsychotics) have a high affinity towards other receptors. Specifically, many of them show a higher affinity towards serotonin 5-HT_2A_ receptors than towards D_2_ receptors. It has been hypothesized that this higher 5-HT_2A_/D_2_ affinity ratio contributes to the lower incidence of side effects of atypical antipsychotic drugs: EPS and prolactin elevation (3). Binding to 5-HT_2A_ receptors could theoretically also lead to improved efficacy towards negative symptoms in schizophrenia (4). 5-HT_2A_ antagonism may confer atypicality on antipsychotic drugs with relatively weaker D_2_ antagonism (or partial D_2_ agonism) because of the ability of 5-HT_2A_ receptors to modulate the activity of dopaminergic neurons differentially in different regions of the brain (5).

Predicting human receptor occupancy in a quantitative manner based on animal studies is one of the challenges in the drug discovery and development process. Pharmacokinetic and pharmacodynamic (PK-PD) modeling tools are extensively used to describe drug distribution and effect (6). Recently utilization of mechanistic factors in the PK-PD modeling has been strongly advocated (7). Inclusion of mechanistic factors like biophase distribution and receptor association and dissociation kinetics allows for a better understanding of processes leading to the observed data (8), as well as distinguishing between system- and drug-specific parameters and extrapolation of drug effects from rat to human (7).

The aim of this study was to develop a population PK-PD model describing D_2_ RO for the atypical antipsychotics risperidone and paliperidone in rats. As a starting point we used a hybrid physiologically-based PK-PD model which has recently been published for the atypical antipsychotic drug olanzapine in rats (9). This model describes the plasma pharmacokinetics using conventional compartmental analysis techniques while processes in the brain are described in a more physiological manner, taking into account the distribution of a drug in the brain and association and dissociation kinetics at the D_2_ receptors. Here, we apply this model to other antipsychotics: risperidone and 9-hydroxy-risperidone (paliperidone). Both drugs are atypical antipsychotics with high affinity for D_2_ and 5-HT_2A_ receptors. Risperidone is metabolized to paliperidone and both drugs show similar binding properties and clinical effect profiles. Therefore, to properly describe the RO of risperidone and eventually its clinical effects it is necessary to take into account the formation of paliperidone, its distribution to the brain and its binding to receptors. Therefore, we extended the previously published model to incorporate metabolite formation, its brain kinetics and its receptor binding parameters. Also, since both risperidone and paliperidone are known P-glycoprotein (P-gp) substrates (10) we included an active efflux process at the blood-brain- barrier. We also investigated whether binding to 5-HT_2A_ receptors influenced the PK and PD of both drugs.

## METHODS

### Data

This work was performed within the framework of the Dutch Top Institute Pharma project: Mechanism-based population PK–PD modeling (http://www.tipharma.com). This modeling platform involves leading global pharmaceutical companies and academic institutions from The Netherlands. Data used for the modeling were generated previously by the pharmaceutical companies: Janssen Research and Development (Belgium), Merck Sharp & Dohme Limited (The Netherlands) and Pfizer Global Research and Development (USA) and were anonimized before releasing to the modelers. Results from a number of studies were used including dose-response and time course studies. Male Sprague Dawley or Wistar rats were used for the experiments. Risperidone (RIS) was administrated intravenously (IV), intraperitoneally (IP) or subcutaneously (SC) in a wide range of single doses (0.01 to 40 mg/kg). In most experiments, RIS plasma and brain concentrations and its RO (either D_2_ or 5-HT_2A_) were measured in one animal at one time point (since animals have to be euthanized for brain concentration and RO measurements). In a few studies paliperidone (PALI) concentrations were measured after RIS or PALI administration. An overview of the studies utilized is given in Table I. For RO studies either the striatum or the frontal cortex was removed for the measurement of D_2_ or 5-HT_2A_ receptor occupancy, respectively. The rest of the brain was homogenized and drug concentration was measured. An *in vivo* or *ex vivo* method was used for both 5-HT_2A_ and D_2_ RO measurements. [^3^H]raclopride was used as a radioligand for D_2_ RO studies and [^3^H]M100907 for 5-HT_2A_ RO studies. The experimental procedures for the plasma sample collection, brain dissection, tissue homogenization and RO measurements were similar across the different study sites and these procedures were based on published reports (11,12).

**Table I:** Overview of Available Data Used in the PK-PD Analysis

Study #	# of rats	drug^1^	ROA^2^	dose [mg/kg]	time points [h]	observation type	RO method^3^
1	3	RIS	IV	2.5	0.12, 0.33, 1, 2, 4, 8	PC-R	NA
2	4	RIS	IV	2	0.12, 0.33, 1, 2, 4	PC-R	NA
3a	23	PALI	SC	5	0.25, 0.5, 1, 2, 4, 8	PC-P, StrC-P, CorC-P	NA
3b	23	RIS	SC	5	0.25, 0.5, 1, 2, 4, 8	PC-R, PC-P, StrC-R, StrC-P, CorC-R, CorC-P	NA
4	42	RIS	IP	0.3, 3	0.25, 0.5, 1, 1.5, 2, 4, 7	PC-R, PC-P, BC-R, BC-P	NA
5	20	RIS	IP	1	0.25, 0.5, 1, 1.5, 2	PC-R, BC-R, RO-D_2_	*in vivo*
6	23	RIS	IP	0.01, 0.03, 0.1, 0.3, 1, 3	1	PC-R, BC-R, RO-D_2_	*in vivo*
7	36	RIS	SC	0.16, 10	0.5, 1, 2, 4, 8, 24	PC-R, BC-R, RO-D_2_	*ex vivo*
8a	12	PALI	SC	0.16, 0.63, 2.5, 10	1	RO-D_2_	*in vivo*
8b	18	RIS	SC	0.04, 0.16, 0.63, 2.5, 10, 40	1	RO-D_2_	*in vivo*
9	19	RIS	IP	0.01, 0.03, 0.01, 0.3, 1	1	PC-R, BC-R, RO-5HT_2A_	*in vivo*
10	20	RIS	IP	0.1	0.25, 0.5, 1, 1.5, 2	PC-R, BC-R, RO-5HT_2A_	*in vivo*
11	24	RIS	IP	0.01, 0.03, 0.01, 0.3, 1, 3	1	PC-R, RO-5HT_2A_	*ex vivo*
12	15	RIS	IP	0.1, 0.3, 0.8, 1	1	PC-R, RO-5HT_2A_	*in vivo*

*RIS* risperidone, *PALI* paliperidone, *IV* intravascular, *IP* intraperitoneal, *SC* subcutaneous, *PC-R* RIS plasma concentration, *PC-P* PALI plasma concentration, *BC* brain concentration, *StrC* striatum concentration, *CorC* cortex concentration, *RO* receptor occupancy, *NA* not applicable

^1^drug administered

^2^route of administration

^3^method used for RO measurement

### Model Development

We used a population approach to describe the pharmacokinetics and pharmacodynamics (receptor binding) of RIS and PALI and to obtain population parameter estimates together with the inter-individual variability. Modeling was done using the non-linear mixed effects modeling software NONMEM (version VII level 2) (13). ADVAN 13 subroutine was used to allow explicit writing of differential equations describing receptor dynamics (Appendix). Log-transformed plasma and brain drug concentrations were used for the data analysis, and concentrations below the limit of quantification were excluded from this analysis.

Inter-individual variability (IIV) on each parameter was modeled assuming a log-normal distribution. Additive, proportional and combined residual error models were tested.

A number of structurally different PK and PD models have been evaluated (see below). Model selection was based on the likelihood ratio test, parameter estimates and their relative standard errors, residual error values and goodness-of-fit plots. An additional structural parameter or inter-individual variability was included in the model, if the resulting change in objective function value (ΔOFV) was >6.64 (*p* < 0.01 assuming χ^2^ distribution). The following goodness-of-fit plots were inspected visually in order to assess the fit of the model to the data: observations *versus* population and individual predictions, individual weighted residuals *versus* individual predictions and conditional weighted residuals (CWRES) *versus* time.

During the analysis, Pirana software (14) was used for NONMEM run management and Xpose package in R (15,16) for making diagnostic plots.

### General Model Structure and Modeling Approach

We did a sequential analysis where we first described the plasma PK of RIS and PALI in a conventional compartmental way. We assumed that this analysis provides an adequate description of concentration in plasma, which in turn is the driving concentration for the brain distribution and receptor binding model. Therefore, it is appropriate to fix plasma parameters while simultaneously estimating brain distribution and receptor binding parameters from brain concentration and receptor occupancy data. This has been done in the second part of the analysis.

### Plasma PK Model

**Fig. 1:**
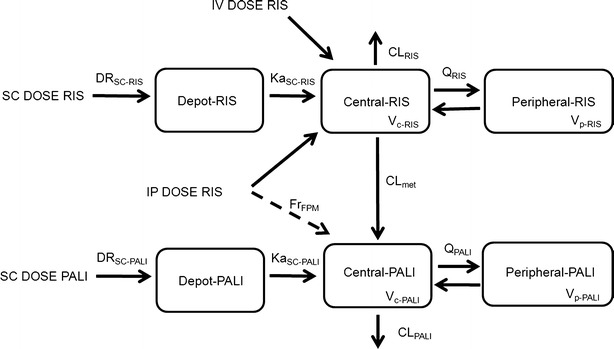
A schematic representation of the plasma PK model. Plasma PK of both RIS and PALI follows a two-compartment model. IV and IP dosing goes directly to the central compartment. A fraction of the absorbed dose for IP RIS route of administration goes directly to the RIS central compartment and a fraction of the absorbed dose goes to the PALI central compartment (Fr_FPM_) representing first pass metabolism. Absorption after SC dosing is described by consecutive zero- and first order processes for both RIS and PALI. DR_SC_ is the duration of the zero-order process after SC dosing. Total elimination clearance of RIS is divided into metabolic clearance (CL_met_) and the clearance by other routes of elimination (CL_RIS_).

One- and two-compartment models for both RIS and PALI were tested. For SC and IP administration different absorption models were tested: zero-order and first-order absorption in combination with a lag time. For IP dosing of RIS we checked models with and without first pass metabolism by assuming that part of the administered RIS dose is converted to PALI before reaching the systemic circulation. Bioavailability for IP and SC doses were estimated relative to IV dosing. Since there were no data after IV administration of PALI, we assumed that RIS and PALI have the same bioavailability for the SC route of administration. This allows estimating the volume of distribution and other absorption parameters of PALI. To account for metabolite formation we divided the elimination clearance for RIS into two clearances: metabolic clearance to PALI (CLmet) and clearance by other routes of elimination (CL_RIS_) (Fig. 1). First-order conditional estimation method (FOCE) was used to obtain PK parameter estimates.

### PK-PD Modeling

After finding the appropriate plasma PK model, population parameters for plasma (mean and inter-individual variability) were fixed after which brain concentrations and RO were fitted simultaneously. Initially only D_2_ RO was taken into account as D_2_ binding is assumed to be crucial for the drug’s antipsychotic action (2). We started with the previously published hybrid physiologically-based PK-PD model (9), but also checked simplifications of this model, i.e. binding not affecting brain kinetics (simplified model from (9)) and reduction in the number of brain compartments by merging intra- and extra-vascular compartments together or assuming only one compartment for drug not bound to receptors in the brain.

**Fig. 2:**
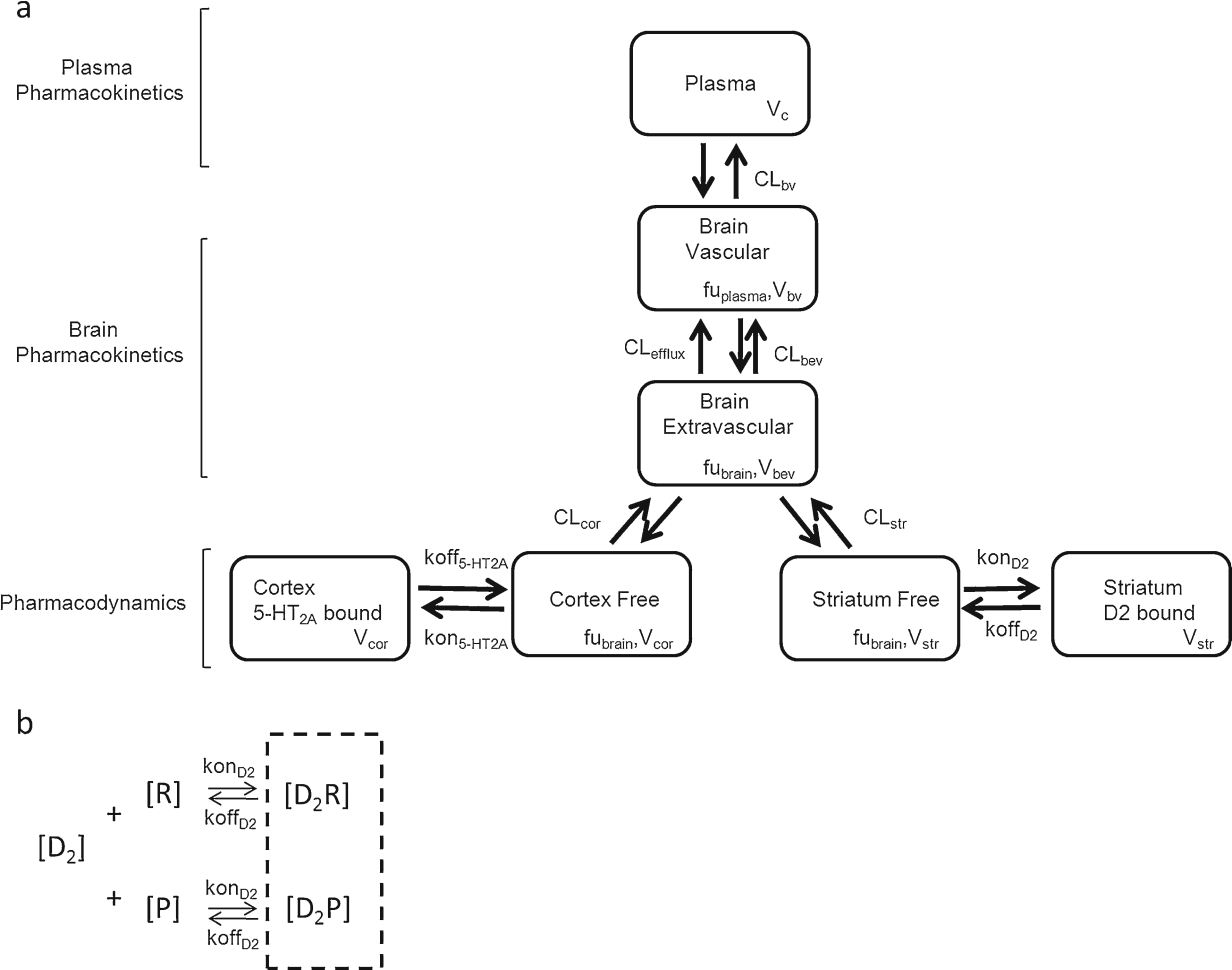
(**a**) A schematic representation of the PK-PD model. The plasma PK has been omitted (see Fig. 1) and brain kinetics and receptor binding have been presented here for one drug only because of the complexity of the model. The same model structure applies for RIS and PALI. (**b**) Representation of the competitive binding to the same receptors by RIS and PALI. Measured RO is the sum of occupancies obtained by both drugs. Here only binding to D_2_ receptors is shown. The same principle applies for 5-HT_2A_ receptors. [D_2_] - concentration of free D_2_ receptors, [R] – unbound concentration of RIS, [D_2_R] - concentration of D_2_ receptor complex with RIS, [P] – unbound concentration of PALI, [D_2_P] - concentration of D_2_ receptor complex with PALI. D_2_ receptor occupancy (RO) is the sum of RO exerted by both drugs.

The hybrid physiology-based PK-PD model consists of four compartments in brain: vascular, extra-vascular, striatum free and striatum bound compartment (Fig. 2). Volumes of these compartments were fixed to physiological values: 0.00024, 0.00656, 0.0002 L/kg for vascular, total extra-vascular and striatum, respectively (17,18). Clearance between plasma and vascular compartment (CL_bv_) was assumed to be equal to cerebral blood flow in rats, which is 0.312 L/h/kg (17), for both RIS and PALI. In the model, transport of RIS and PALI between the vascular and extra-vascular compartment across the blood-brain barrier (BBB) was governed by two processes: passive diffusion and active efflux. Separate values of passive clearance (CL_bev_) and active efflux clearance (CL_efflux_) were estimated for RIS and PALI when possible. We checked whether linear or saturable efflux processes described the data best. Only unbound drug could cross the brain-blood barrier (BBB). Plasma protein binding is constant over wide range of concentrations in humans (19). We assumed that the same is true for rats and plasma and brain fraction unbound were fixed to literature values: fu_plasma-RIS_ = 0.0798, fu_brain-RIS_ = 0.0699, fu_plasma-PALI_ = 0.129, fu_brain-PALI_ = 0.0755 (20).

We assumed fast equilibration between the extra-vascular and striatum free compartments. This was achieved by fixing the corresponding clearance (CL_str_) to a high value (500 L/h/kg). In striatum, RIS and PALI reversibly bind to D_2_ receptors. Binding to receptors was described using kon as the receptor association rate constant (nM^−1^ h^−1^), koff as the receptor dissociation rate constant (h^−1^), and B_max_ as the maximum binding capacity of these drugs to the receptor. Including explicit binding kinetics is justified because of the hysteresis observed between D_2_ (or 5-HT_2A_) receptor occupancy and brain concentration when excluding striatum or frontal cortex (from D_2_ and 5-HT_2A_ occupancy studies, respectively; data not shown). RIS and PALI compete for the same receptors and hence the measured RO is the sum of occupancies obtained by both drugs.

Both RIS and PALI have a strong affinity towards 5-HT_2A_ receptors. Therefore, we decided to evaluate also an extended model in which we included binding to these receptors in the frontal cortex, where the density of 5-HT_2A_ receptors is the highest (21). To that end, we included two additional compartments: cortex free and cortex bound. We fixed the volume of frontal cortex to 0.0035 L/kg (22). As for striatum, we assumed fast equilibration between brain extra-vascular and cortex free compartments. Binding to 5-HT_2A_ receptors was described using kon, koff and B_max_ values specific for these receptors. Kd (dissociation rate constant) and koff were estimated from the model and kon was calculated as koff/Kd. We checked whether we could estimate different binding constants for data obtained from *in vivo* and *ex vivo* binding experiments.

First-order conditional estimation method (FOCE) was used to fit the models.

### Model Evaluation

For the plasma PK model a bootstrap analysis was done to determine the precision of the parameter estimates. In the bootstrap technique, bootstrap replicates are generated by randomly sampling individuals from the original dataset with replacement. 1000 samples were used and they were stratified by study. Parameter estimates for each of the re-sampled datasets were obtained by fitting the final plasma PK model using NONMEM. Median, 5-th and 95-th percentiles were calculated for all the parameters and medians of bootstrap estimates were compared with parameter values obtained from the original dataset.

Additionally, since the original dataset is rather heterogeneous and bootstrapping may lead to biased results, we also did log-likelihood profiling (LLP). In this method each parameter is in turn fixed to lower or higher values than the one estimated by the model and the reduced model is fit to the data. Obtained OFV is compared with the OFV of the original model. The 90% confidence interval (CI) of a parameter is calculated by finding the value of the parameter at which the difference in OFV is 3.84 (*p* = 0.05 assuming χ^2^ distribution). Bootstrap analysis and log-likelihood profiling were done with the help of the software package Perl Speaks NONMEM (PsN) (23).

Due to the great heterogeneity of the dataset and very long run times we did not perform a bootstrap analysis for the PD model. However, we did the log-likelihood profiling to find 90% CIs of the parameter values.

In order to check the predictive performance of the model we simulated 1000 datasets from the final PK-PD model. Then we graphically compared the observed plasma and brain concentrations and D_2_ and 5-HT_2A_ RO with median and 90% prediction intervals calculated from the simulated data for each dose and route of administration separately.

### Brain-To-Plasma Ratios

We simulated brain and plasma concentrations based on population parameter estimates (without inter-individual and residual variability) for doses and time points corresponding to the ones seen in the data set. For each simulated time point we calculated brain-to-plasma ratios and compared them graphically with the observed brain-to-plasma ratios, plotting only brain-to-plasma values if both plasma and brain concentrations were above the level of quantification. To check the influence of different parameters, we also simulated the brain-to-plasma ratio pattern in the absence of active efflux and with a 10 times higher value of brain clearances and of increases or decreases of the receptor association and dissociation constants kon and koff. To that end, we either fixed CL_efflux_ to zero or used CL_bev_ and CL_efflux_ 10 times higher than estimated from the model, or kon/koff values 10 times lower or higher than estimated by the model. All other parameter values were the same as estimated by the model. We used R software for the simulations.

## RESULTS

### Plasma PK

Plasma PK was best described by a two-compartment model for both RIS and PALI (Fig. 1). For the SC route of administration consecutive zero- and first-order absorption described the data best. For the IP route absorption parameters could not be estimated and it was assumed that dosing was directly to the central compartment. However, including first pass metabolism where a fraction of the RIS dose goes directly to the PALI central compartment improved the fit of the model. Parameter estimates of the final model are given in Table II. In the final model six inter-individual variability parameters (for F_IP_, Ka_SC-RIS_, Ka_SC-PALI_, DR_SC-RIS_, CL_RIS_, CL_PALI_) were retained.

**Table II:** Parameter Estimates of the Plasma PK Model from the Original Dataset and from 1,000 Bootstrap Replicates

Parameter	Parameter estimate (% RSE)	Bootstrap median	5th-95th percentile from bootstrap	90% CI obtained from log-likelihood profiling
F_IP-RIS_	0.412 (12)	0.413	0.314–0.540	0.321–0.530
F_SC_	0.816 (9)	0.810	0.654–1.03	0.672–0.987
Fr_FPM_	0.268 (12)	0.271	0.213–0.337	0.209–0.332
Ka_SC-RIS_ (1/h)	2.84 (20)	2.90	2.25–4.07	2.13–4.16
Ka_SC-PALI_ (1/h)	1.31 (22)	1.31	1.00–1.77	0.969–1.84
DR_SC-RIS_ (h)	0.161 (55)	0.170	0.0157–0.313	0.0490–0.283
DR_SC-PALI_ (h)	0.162 (54)	0.167	0.0848–0.261	0.0276–0.320
V_c-RIS_ (L/kg)	1.29 (6)	1.28	1.07–1.56	1.11–1.49
CL_RIS_ (L/h/kg)	1.62 (9)	1.62	1.29–2.07	1.34–1.95
CL_met_ (L/h/kg)	0.775 (11)	0.757	0.591–0.974	0.623–0.961
V_p-RIS_ (L/kg)	0.169 (16)	0.168	0.128–0.223	0.131–0.220
Q_RIS_ (L/h/kg)	0.0882 (25)	0.0891	0.0529–0.137	0.0601–0.132
V_c-PALI_ (L/kg)	1.27 (15)	1.21	0.0647–1.64	0.0087–1.66
CL_PALI_ (L/h/kg)	1.06 (10)	1.04	0.768–1.36	0.847–1.32
V_p-PALI_ (L/kg)	0.251 (54)	0.281	0.119–2.11	0.0767–75.5
Q_PALI_ (L/h/kg)	0.269 (128)	0.245	0.0428–23.4	0.0294–48.3
Inter-individual variability				
IIV-FIP (%CV)	80.6 (7.7)	80.4	70.8–89.7	70.0–92.9
IIV-Ka_SC-RIS_ (%CV)	46.4 (52.3)	47.9	29.5–70.5	27.1–84.3
IIV-Ka_SC-PALI_ (%CV)	34.4 (30.2)	33.0	11.12–50.6	17.0–58.7
IIV-DR_SC-RIS_ (%CV)	91.2 (80.6)	84.2	0.912–287	17.3–211
IIV-CL_RIS_ (%CV)	30.5 (13.3)	29.7	16.3–38.2	23.2–39.0
IIV-CL_PALI_ (%CV)	16.2 (32.8)	16.0	10.4–22.6	10.9–24.5
Proportional residual error				
Risperidone	0.233 (16.5)	0.220	0.174–0.279	0.192–0.288
Paliperidone	0.186 (14.2)	0.171	0.131–0.214	0.147–0.238

% RSE - Relative Standard Error as calculated by NONMEM covariance step

Inter-individual variability is expressed as percent coefficient of variation

Parameters for the rate of zero-order absorption and volume of distribution and inter-compartmental clearance for PALI could not be estimated precisely (Table II). However, we decided to keep them in the model since removing them led to a significantly worse fit and it was important to describe the plasma PK as precisely as possible for the description of brain kinetics and receptor binding.

**Fig. 3:**
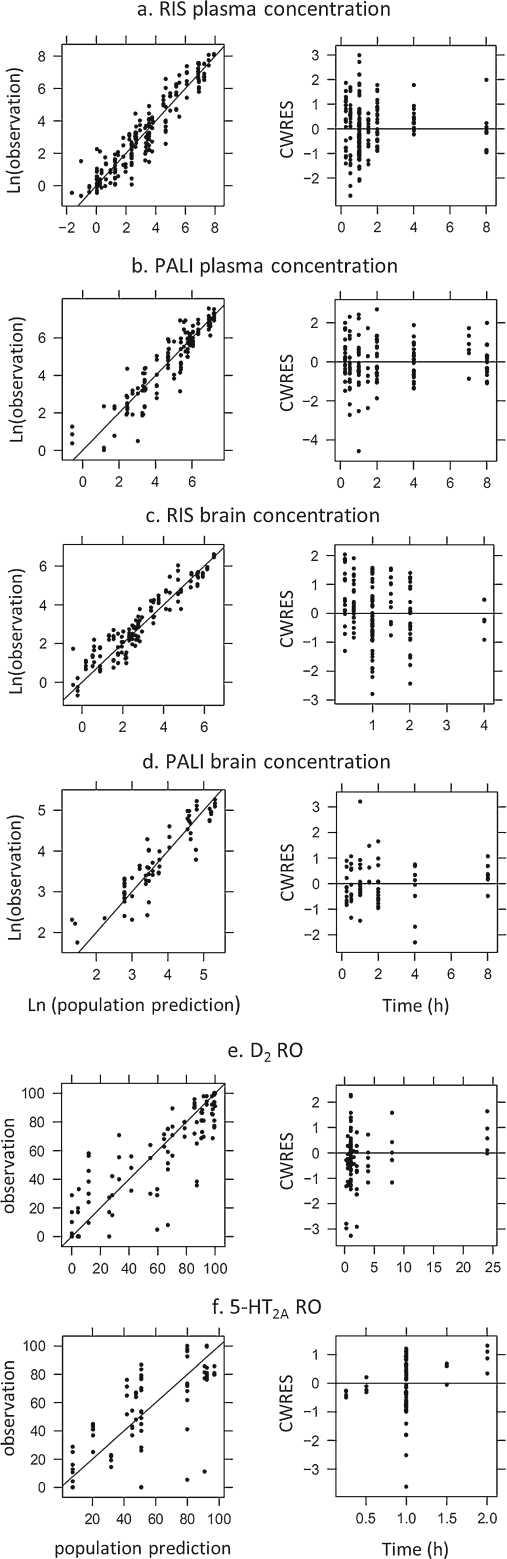
Goodness-of-fit plots of the PK-PD model. Presented are scatter plots of plasma and brain concentrations and D_2_ and 5-HT_2A_ RO versus population predictions and conditional weighted residuals (CWRES) versus time.

Goodness-of-fit plots did not show any systematic deviation between observations and population and individual predictions nor any trends in conditional weighted residuals *versus* time, which demonstrates that this model adequately describes the plasma PK of RIS and PALI (Fig. 3).

### PK-PD

The previously published hybrid physiology-based PK-PD model (9) fitted the data best after adjusting for binding to 5-HT_2A_ receptors (Fig. 2). A model with only D_2_ receptor binding (9) led to high residual error (>60%) for brain concentration. Using a combined error model reduced the proportional error to some extent. However, this model did not explain the observed brain-to-plasma ratio adequately (Fig. 6a–b). These problems were overcome when the model was extended to include 5-HT_2A_ receptor binding in frontal cortex.

Our data did not allow us to estimate reliably all brain clearance parameters (CL_bev-RIS_, CL_efflux-RIS_, CL_bev-PALI_, CL_efflux-PALI_). Change in parameter values within relatively wide range did not lead to changes in model fit. Therefore, we assumed that CL_bev_ or CL_efflux_ is the same for RIS and PALI. Assuming a single CL_efflux_ parameter led to termination problems. However, the model with a single CL_bev_ value had an OFV only slightly lower (<0.5) than the model with four clearance parameters and RSE values obtained from the covariance step were acceptable. Therefore, in the final model CL_bev_ was assumed to be the same for RIS and PALI.

Different Kd and koff values for D_2_ and 5-HT_2A_ were estimated by the model. However, we were not able to estimate different Kd and koff values for RIS and PALI, therefore we assumed that Kd and koff values are the same for RIS and PALI, which is consistent with literature *in vitro* values (24). Similarly, we also had to assume that the binding rate constants for both *in vivo* and *ex vivo* binding RO measurements were the same. Describing active efflux from brain as a saturable process did not improve the fit therefore in the final model the active efflux was assumed to be linear. Due to the complexity of the model we were not able to estimate IIV variability for the brain PK-PD model parameters. Therefore, we fixed the IIV parameters to zero. Final parameter estimates of the model are given in Table III.

**Table III:** Parameter Estimates of the PK-PD Model and Their Relative Standard Error

Parameter	Parameter estimate	% RSE	90% CI obtained from log-likelihood profiling
CL_bev-RIS_ (L/h/kg)	2.13	29	0.333–>1000^c^
CL_bev-PALI_^a^(L/h/kg)	2.13	29	0.333–>1000^c^
CL_efflux-RIS_ (L/h/kg)	9.97	28	1.49–>1000^c^
CL_efflux-PALI_ (L/h/kg)	47.0	28	7.24–>1000^c^
Kd_D2_ (nM)	0.463	14	0.336–0.628
koff_D2_ (1/h)	0.671	19	0.427–1.03
kon_D2_^b^ (1/nM/h)	1.45	–	–
B_max-D2_ (nM)	245	15	194–305
Kd_5-HT2A_ (nM)	0.219	15	0.134–0.313
koff_5-HT2A_ (1/h)	0.525	25	0.257–0.970
kon_5-HT2A_^b^ (1/nM/h)	2.40	–	–
B_max-5-HT2A_ (nM)	46.5	11	37.3–58.9
Residual error			
proportional BC RIS	0.362	7	0.315–0.419
proportional BC PALI	0.424	18	0.351–0.519
additive % D_2_ RO	17.7	8	15.1–20.7
additive % 5-HT_2A_ RO	18.2	7	1.49–22.4

% RSE - percent Relative Standard Error as calculated by NONMEM covariance step

*BC* brain concentration

^a^assumed to be the same as CL_bev-RIS_

^b^calculated as kon=koff/Kd

^c^OFV does not change with increasing parameter value

Goodness-of-fit plots did not show any systematic deviation between observations and population and individual predictions nor trends in CWRES *versus* time, which demonstrates that this model adequately describes the brain concentrations of RIS and PALI and their D_2_ RO (Fig. 3). Some time course pattern can be seen in the plot of CWRES *versus* time for 5-HT_2A_ RO, but CWRES values are relatively low.

### Model Evaluation

Median bootstrap parameter estimates of the PK model were in good agreement with model estimated population values (Table II). 90% confidence intervals were large for some parameters, especially related to absorption and for the PALI peripheral compartment.

Results of log-likelihood profiling for brain parameters show that most parameters are estimated precisely with exception of the brain clearance parameters (Table III). Further inspection of results showed that the values of clearance parameters (CL_bev_, CL_efflux-RIS_ and CL_efflux-PALI_) did not influence model fit and other parameter estimates much. The upper limits of CI for these parameters could not be found, showing that the transport is fast and equilibration between brain and plasma is not much affected by the exact values of clearances. Moreover, close inspection of the results show that relative value of active efflux clearance to passive clearance seems to be very constant. For all the values of CL_bev_, CL_efflux-RIS_ and CL_efflux-PALI_ inspected by LLP, the ratio of CL_efflux-RIS_ to CL_bev_ was in range of 4.57-4.87 while ratio of CL_efflux-PALI_ to CL_bev_ was in range of 21.1-22.2.

**Fig. 4:**
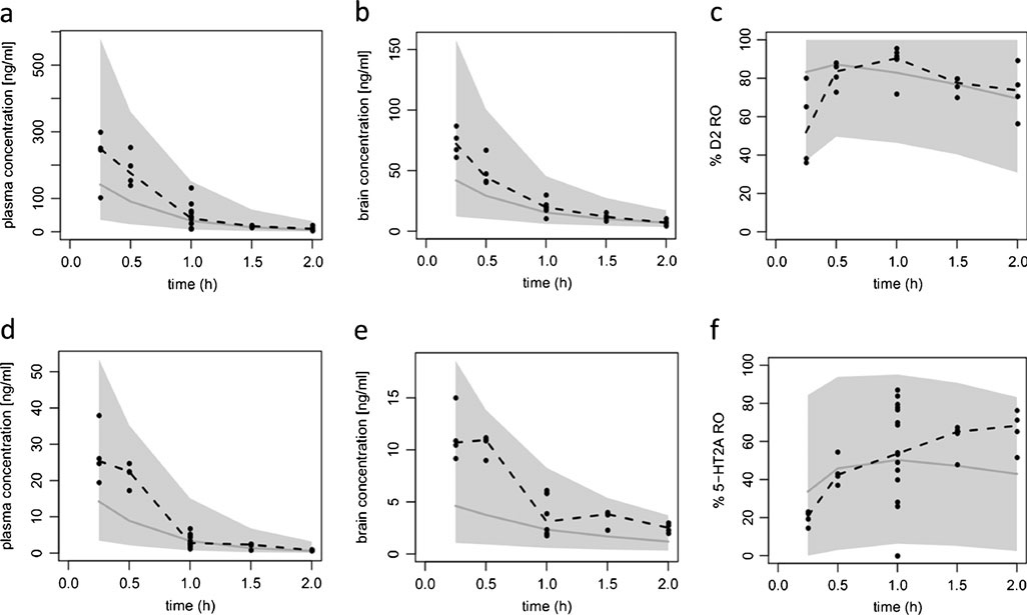
Predictive check of the PK-PD model. (**a**–**c**) Risperidone plasma concentration, risperidone brain concentration after removing striatum and D_2_ RO after IP administration of a 1 mg/kg dose of risperidone, respectively. (**d**–**f**) Risperidone plasma concentration, risperidone brain concentration after removing frontal cortex and 5-HT_2A_ RO after IP administration of a 0.1 mg/kg dose of risperidone, respectively. Dots represent the observed data; the dashed line represents the median of the observed data; the shaded area represents 90% prediction interval based on 1000 simulated datasets; the grey line represents the median of the simulated data.

Some of the predictive check results are depicted in Fig. 4. We present the result for the IP route of administration for doses of 0.1 and 1 mg/kg since for these doses there were time course data available for 5-HT_2A_ and D_2_ RO and they had more data points than other doses. Practically all observations fall within the range of the 5-th and 95-th percentile. Median time course of D_2_ RO is predicted well. For 5-HT_2A_, RO seemed to be underestimated for later time points. Prediction intervals are very wide since the residual error in our model is also relatively big. However, it should be noted that the variability of the data is also large (see for example 5-HT_2A_ RO at 1 h).

Inspection of the model-predicted plasma and brain concentrations for the cases where observations were reported to be below the level of quantification (LOQ), shows that exclusion of the observations below LOQ from the analysis did not lead to a significant bias. For the studies where LOQ was known, only around 15% of observations were above LOQ and in less than 5% of the cases in total predicted concentrations were outside the confidence intervals based on the residual standard error. For the studies where LOQ was not known the predicted concentrations were also low.

### Brain-To-Plasma Ratios

**Fig. 5:**
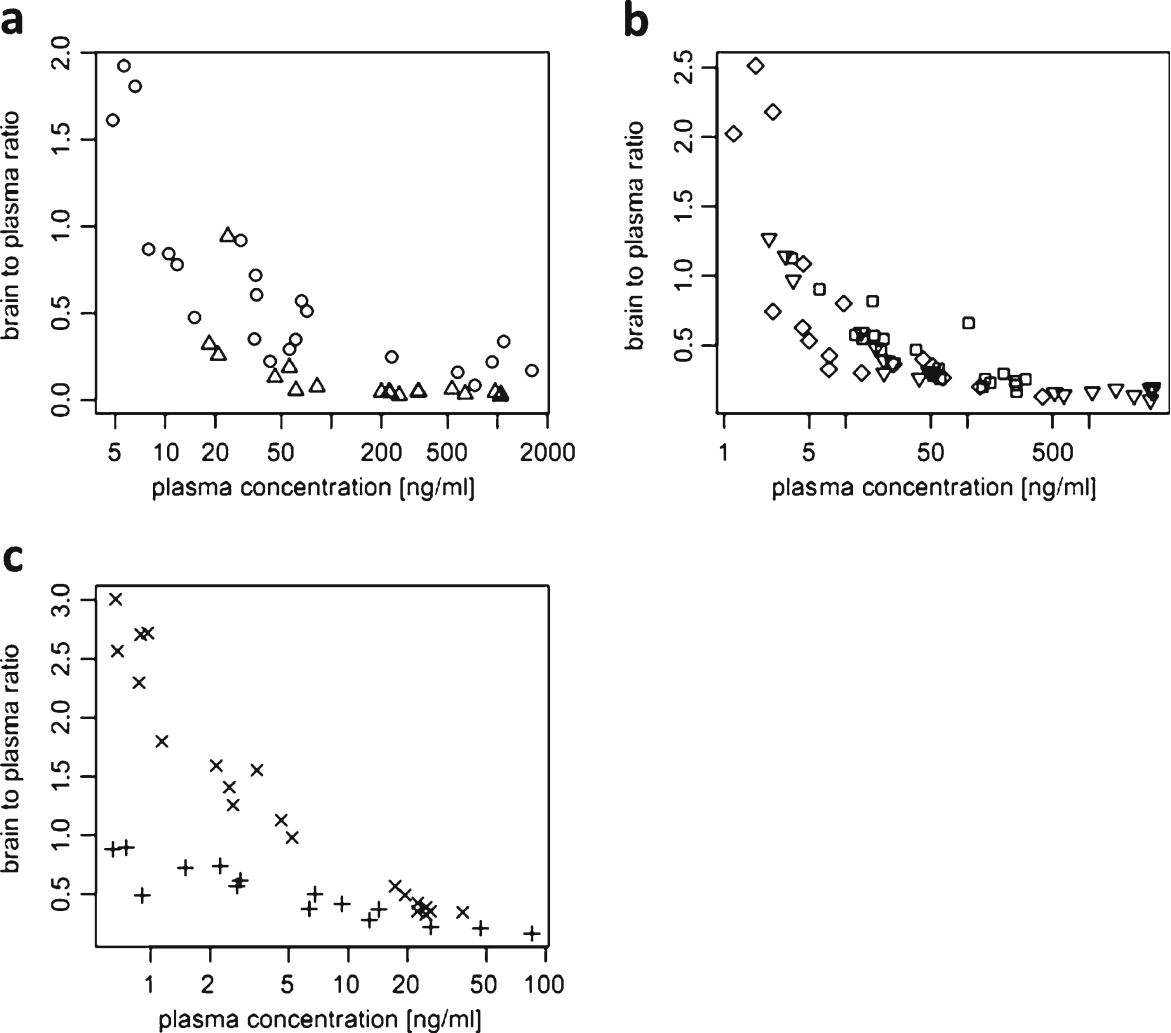
Brain-to-plasma ratios against plasma concentrations. (**a**) Data from studies where total brain concentration was measured; circles - RIS, triangles -PALI. (**b**) Data from D_2_ RO studies where brain concentration was measured after removing striatum. (**c**) Data from 5-HT_2A_ RO studies where brain concentration was measured after removing frontal cortex. For b and c only RIS data was available and different symbols represent different studies.

**Fig. 6:**
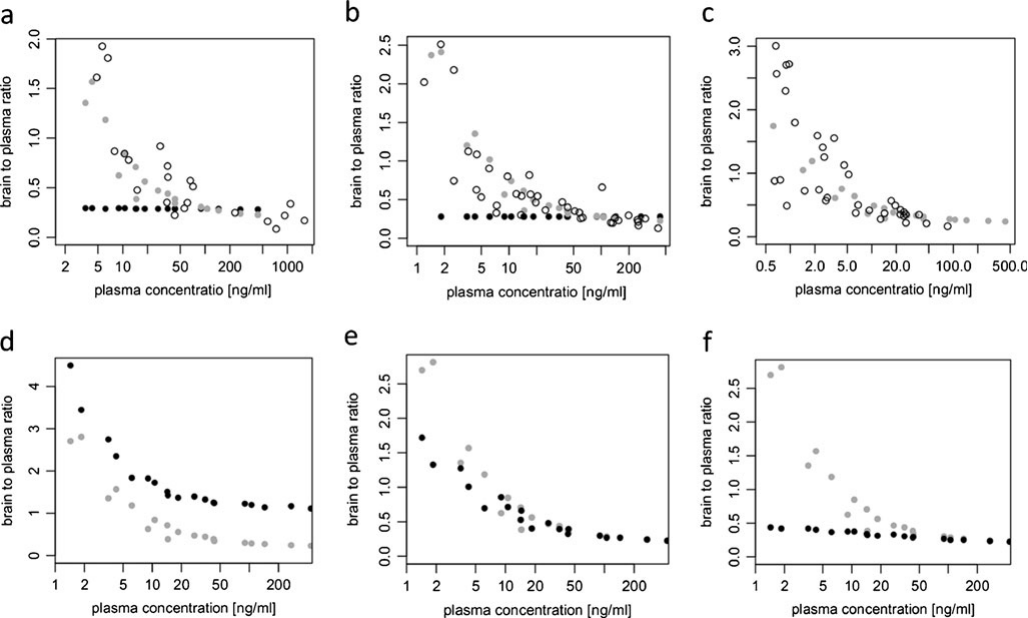
Observed and simulated brain-to-plasma ratios. Open circles in panels a-c represent observed brain-to-plasma ratios for total brain (**a**), brain excluding striatum - from D_2_ RO studies (**b**) and brain excluding frontal cortex - from 5-HT_2A_ RO studies (**c**). In all the panels gray dots represent predictions of our final model. Black dots represent prediction of the model with only D_2_ receptor binding (**a–b**), or prediction of final model but assuming no efflux (**d**), kon and koff values 10 times higher (**e**) or koff values 10 times higher (**f**) than in the final model. Only total brain-to-plasma ratios are depicted in panels d-f. Qualitatively similar results were obtained for brain concentrations from D_2_ and 5-HT_2A_ studies.

The observed brain-to-plasma ratios were higher at lower plasma concentrations and even out as plasma (or brain) concentration increases, both for RIS and PALI (Fig. 5). Even after multiplication of the brain-to-plasma ratio by fu_brain_/fu_plasma_ = 0.876 and obtaining “free brain-to-plasma ratio”, the brain-to-plasma ratio at higher concentrations is lower than one due to active efflux from the brain. This brain-to-plasma ratio pattern was seen for both the total brain concentration and the concentration measured in brain excluding striatum (from the studies where D_2_ RO was measured) or excluding frontal cortex (from studies where 5-HT_2A_ RO was measured). A model with only D_2_ receptor binding in striatum did not predict higher brain-to-plasma ratios for lower concentrations (Fig. 6a–b). Including binding to 5-HT_2A_ receptors in the model predicted brain-to-plasma ratios well (Fig. 6a–c) over the entire concentration range.

Fixing CL_efflux_ to zero led to an increase of brain-to-plasma ratios, but the general pattern stayed the same (Fig. 6d). Increasing CL_bev_ and CL_efflux_ had virtually no effect on the brain-to-plasma ratios (not shown). If koff and kon values for both D_2_ and 5-HT_2A_ receptors were 10 times higher (implying no change in Kd) then brain-to-plasma ratios at lower concentrations were slightly lower and at higher concentrations they were the same as in the original model (Fig. 6e). The same effect was visible with and without efflux. Increasing Kd by increasing koff leads to an almost constant brain-to-plasma ratio (Fig. 6f). Increasing Kd by decreasing kon had a similar effect, but less pronounced (data not shown). If we increased koff for D_2_ receptors only, then brain-to-plasma ratios became constant only for brain concentrations excluding cortex (brain concentration measured in 5-HT_2A_ RO studies; data not shown). If we increased koff only for 5-HT_2A_ receptors then brain-to-plasma ratios became constant only for brain concentrations excluding striatum (brain concentration measured in D_2_ RO studies; data not shown).

## DISCUSSION

In this paper we present an extension of the previously published PK-PD model for olanzapine (9) to two other antipsychotic drugs: risperidone and paliperidone. This model describes the time course of D_2_ receptor occupancy after administration of the antipsychotics and takes brain distribution into account. Here, we expand the model by taking into account metabolite formation and its receptor binding, active efflux from the brain and binding to 5-HT_2A_ receptors.

Some antipsychotic drugs (such as risperidone and aripiprazole) have active metabolites. The active metabolite of risperidone, paliperidone, achieves high concentrations in plasma and brain (25,26). It also shows potent binding to D_2_ and 5-HT_2A_ receptors and is by itself a potent antipsychotic drug (27,28). Therefore, to describe receptor occupancy of a parent drug in a mechanistic way, it is necessary to take into account formation, brain kinetics and receptor binding of the metabolite.

It has been shown in *in vitro* studies and studies with knockout mice, that both risperidone and paliperidone are P-gp substrates (10). Inclusion of active efflux at the BBB in the model was indeed necessary to describe the data properly. Relative values of model predicted parameters for active efflux at the BBB for both drugs are in line with previous experimental studies. Most of the studies with mdr1a knockout mice (10,29) show that the difference in brain-to-plasma ratios between the knockout and the wild-type mice is considerably larger for paliperidone in comparison to risperidone (with one exception where the ratio is slightly higher for risperidone (30)). Similar results have been found in *in vitro* experiments with MDCK cells in an transwell assay (31) and are in line with our results. It has been assumed in our model that the active efflux is a linear process. This seems to be a valid assumption for risperidone and paliperidone, since modeling the active transport as a saturable process did not improve the fit. This is in line with the finding that the highest brain concentrations of risperidone and paliperidone seen in our dataset were around 2 μM, while the Km values (concentration of substrate yielding half maximum activity) for P-gp transport from *in vitro* studies range between 5.6 to 26 μM (30,32,33). So clearly, the concentrations observed *in vivo* are far below the saturation levels for risperidone and paliperidone.

Paliperidone is less lipophilic than risperidone, therefore one would expect that it will diffuse slower through biological membranes. However, *in vitro* essays show that passive permeability of risperidone and paliperidone are quite similar (31). Therefore, assuming the same value for passive clearance through BBB can be justified. Probably more data, especially for paliperidone, would be needed to estimate separate parameters of both drugs. However, it should be noted that it might be difficult to estimate passive and active clearance parameters reliably. It seems that, at least in our dataset, brain concentration is only informative about the ratio of active and passive processes. Extensive simulation study would be probably necessary to establish what data is needed in order to be able to estimate precisely brain clearance parameters.

Another extension of the previous model developed for olanzapine is the inclusion of binding to 5-HT_2A_ receptors. We included binding to 5-HT_2A_ receptors when we observed that the model with binding only to D_2_ receptors in striatum did not provide a good fit to the data and also after inspecting observed and model predicted brain-to-plasma ratio plots (Fig. 5, 6a–c). The brain-to-plasma ratio was not constant for risperidone, suggesting the influence of specific binding to receptors on the brain kinetics. Similar plots made for olanzapine show that the brain-to-plasma ratio is practically constant and that the brain concentrations of olanzapine are higher than those for risperidone (data not shown). This suggests that olanzapine shows a different pattern of brain kinetics than risperidone and paliperidone. Therefore, we explored the influence of the different parameters on the brain-to-plasma ratio. First, we simulated brain-to-plasma ratios assuming no active efflux, to see if higher brain concentrations would lead to more constant brain-to-plasma ratios. Our simulations show that even without efflux, the brain-to-plasma ratios were not constant (Fig. 6d), suggesting that the higher brain-to-plasma ratio was not related to the active efflux process. Next we investigated whether the pattern could be explained by the disequilibrium between plasma and brain caused by slow transport between the two. However, increasing brain clearance had virtually no effect on brain-to-plasma ratios. We also looked at the influence of receptor binding parameters on the brain-to-plasma ratios. Therefore, we simulated the influence of increasing values for both koff and kon ten-fold, reflecting a more rapid equilibration of receptor binding, without changing the receptor affinity Kd. This resulted in slightly lower brain-to-plasma ratios at the lowest concentrations (Fig. 6e). This suggests that the increased brain-to-plasma ratio may be only partly explained by disequilibrium between unbound and bound drug. Finally, an increase of the Kd values by increasing koff or decreasing kon ten-fold led to more constant brain-to-plasma ratios (Fig. 6f). A higher Kd implies that receptor occupancy is lower at the same unbound brain concentration, and therefore the contribution of the bound drug to the total brain concentration is less pronounced, resulting in a lower brain-to-plasma ratio.

Olanzapine has lower binding affinity for D_2_ and 5-HT_2A_ receptors than risperidone and paliperidone (9,34). According to our model, brain-to-plasma ratios are constant under these conditions. The lower D_2_ binding affinity of olanzapine as compared with risperidone may explain why for olanzapine a simpler model with only binding to D_2_ receptor and binding not affecting brain kinetics could explain the data well (9). Risperidone and paliperidone have relatively low values of the dissociation constant for 5-HT_2A_ receptors compared to other antipsychotics (34,35), so one would expect that a simpler model (where binding to 5-HT_2A_ receptors does not affect brain distribution) could be appropriate for these drugs. Even in cases where kon and koff values are unknown, plotting brain-to-plasma ratios against plasma concentrations would indicate whether a simpler model would be appropriate.

In the model for olanzapine, the receptor density (B_max_) did not influence the model fit and could be removed from the model (9). For risperidone and paliperidone, receptor binding influenced brain kinetics, therefore receptor density was an important parameter. This was demonstrated by the precise estimate of B_max_ for both D_2_ and 5-HT_2A_ receptors (Table III). B_max_ values estimated by the model were 245 nM and 47.0 nM for D_2_ receptors in striatum and 5-HT_2A_ receptors in frontal cortex, respectively. This 5-HT_2A_ receptor density is in line with the values of 25–60 nM estimated from *in vitro* studies (36–39) (assuming 10% protein content (40)). Average D_2_ receptor density from *in vitro* studies is 48 nM (40) with a highest estimated value of 75 nM (41). This is approximately 3 to 5 times lower than the value estimated by the model. However, some discrepancy is not surprising since in our model B_max_ represents the theoretical receptor density, while in *in vitro* assays it represents the density of receptors available for the radioligand used. Fixing B_max_ to values different than the ones estimated by the model worsened the model fit (results not shown). Simulations showed that changing B_max_ values lead to considerable changes in brain concentration and receptor occupancy (data not shown).

To our knowledge, no values are published for *in vivo* or *in vitro* association and dissociation constants for D_2_ or 5-HT_2A_ receptors in rats for risperidone and paliperidone. *In vitro* binding constant (Ki) values (which can approximate Kd values) for D_2_ binding found in literature are usually around 2 to 3 nM (34,42–44), however a value of 0.44 for risperidone has also been reported (45). For 5-HT_2A_ binding *in vitro* Ki values of 0.12–0.39 and 0.25 nM for risperidone and paliperidone, respectively, have been reported (34,42,43,45). Therefore, the *in vivo* values obtained in our model are within the range of *in vitro* values in rat tissues for 5-HT_2A_ binding and somehow on the lower end of the *in vitro* range for D_2_ binding. Difference between values obtained *in vivo* and *in vitro* may be caused by different radioligands used in *in vitro* and *in vivo* studies. It is also conceivable that the receptor-binding properties of drugs in brain neuronal cells under *in vivo* conditions differ significantly from those in brain homogenates and membrane fractions (46).

We assumed the same binding affinities for risperidone and paliperidone, since our data sets did not allow estimation of separate kon and koff values for risperidone and paliperidone. More receptor occupancy data for paliperidone would be necessary to estimate paliperidone association and dissociation rate constants. However, since published *in vitro* Ki values in rat brain tissues are similar for risperidone and paliperidone (34), assuming the same rate constants for both drugs should not lead to much bias, especially since the parameters were estimated quite precisely (RSE between 14 and 25%).

Assuming the same values for binding constants for risperidone and paliperidone may theoretically be one of the reasons why there are relatively big residual errors for brain concentrations and receptor occupancy estimated in our model (Table III). This could also apply to RO measurements obtained from *in vivo* and *ex vivo* studies. Trying to estimate separate binding parameters for *in vivo* and *ex vivo* measurements or excluding the less common *ex vivo* RO data from the analysis resulted in problems with model convergence and in less precise parameter estimates. Use of different rat strains could also theoretically explain at least a part of the intra- and inter-individual variability. However, when we plotted individual post-hoc estimates for different parameters, we did not see any difference between the different rat strains or different RO measurement methods.

Considerable residual errors both in PK and PD may stem partly from differences in experimental procedures (especially brain dissection) on different occasions and at the different pharmaceutical companies that provided the data. Body weight variations could also potentially explain a part of the observed inter-individual variability, but we had no information of body weight of the rats allowing us to use it as a covariate in the model. Residual errors could probably be reduced if there was an inter-individual variability in brain PK-PD parameters. However, we were not able to estimate these variabilities and decided to fix them to zero.

A predictive check of the PK-PD model showed that it can predict D_2_ RO reasonably well up to 8 h (Figs. 3e and 5). But the model seems to lead to under-prediction at 24 h (Fig. 3e). Median 5-HT_2A_ RO also seems to be under-predicted for later time points (Fig. 4). Only two studies had a time course of D_2_ RO and one study a time course of 5-HT_2A_ RO while most of the studies had only observations at the 1 h time point. On the one hand, this unbalanced dataset could lead to parameter estimates which explain all data well, but with some model misspecification for the later time points. On the other hand, with just a few observations for later time points it is difficult to judge if a deviation between observation and prediction is not just a random error. More D_2_ and 5-HT_2A_ RO time course data are necessary in order to obtain better description of the full time course of receptor occupancy by the model.

Our dataset shows that binding to receptors influences brain concentrations and that D_2_ binding alone could not explain the data well. In the model we included binding to D_2_ and 5-HT_2A_ receptors (the only available data), but both risperidone and paliperidone also bind to other receptors. *In vitro* data with rat tissue or rat cloned cells show that risperidone and paliperidone have high affinity (Ki not more than 5 times higher than Ki for D_2_ receptors) for α1 and α2 adrenoceptors and for serotonin 5-HT_7_ receptors (34,44,45). However, densities of all of these receptors in rat brain are relatively low (below 100 fmol/mg of protein) (47–50). Therefore, we would not expect the influence of binding to these receptors on brain kinetics to be significant. Risperidone and paliperidone bind also to histamine H_1_ receptors and other subtypes of dopamine and serotonin receptors (34,45). However, since Ki values for these receptors are more than 5 times higher than Ki values for D_2_ and 5-HT_2A_ receptors, based on our simulations of brain-to-plasma ratio we would not expect this binding to affect the rat brain distribution. Similarly, based on *in vitro* binding to human cloned receptors and receptor densities in the human brain (34,51) binding to other receptors should not strongly influence brain kinetics in human.

Our model can be utilized for the human prediction of D_2_ and 5-HT_2A_ RO. Using the sequential approach, human plasma PK models can be developed separately in a conventional way and receptor occupancy can be predicted afterwards. Usage of a physiology-based approach in describing brain distribution and receptor binding allows utilization of human physiological values, *in vitro* information and rat-to-human scaling to predict human receptor occupancy. This translational approach can also be used for drugs which have an active metabolite or show active efflux at the brain-blood barrier. Since it is known that D_2_ RO is linked with clinical outcome and side effects of antipsychotics (2), but that it is difficult and costly to measure, the ability to predict human D_2_ RO based on plasma data can help with linking different doses of drugs with their clinical effect. This work is ongoing in our research group.

In conclusion, we have shown that the previously published hybrid physiologically-based model structure developed for olanzapine (9) can be utilized to describe the PK-PD of risperidone and paliperidone in rats. However, some drug-specific adjustments were necessary. Addition of active metabolite formation and active efflux was straight-forward. Additionally, binding to 5-HT_2A_ receptors has been included in order to describe the brain distribution well. This may stem from the fact that risperidone and paliperidone have higher affinity to D_2_ and 5-HT_2A_ receptors than olanzapine. Therefore, receptor affinities and brain-to-plasma ratios may need to be considered before choosing the best PK-PD model for centrally active drugs.

## APPENDIX

Differential equations used to describe plasma PK:

$$ {\text{d}}\left( {{{\text{A}}_{{{\text{depot}} - {\text{RIS}}}}}} \right)/{\text{dt}} = {\text{DOS}}{{\text{E}}_{{{\text{SC}} - {\text{RIS}}}}}/{\text{D}}{{\text{R}}_{\text{RIS}}}\left( {{\text{t}} < {\text{D}}{{\text{R}}_{\text{SC--RIS}}}} \right) - {\text{k}}{{\text{a}}_{{{\text{SC}} - {\text{RIS}}}}} * {{\text{A}}_{{{\text{depot}} - {\text{RIS}}}}} $$

$$ \matrix{{*{20}{c}} {{\text{d}}\left( {{{\text{A}}_{{{\text{c}} - {\text{RIS}}}}}} \right)/{\text{dt}} = {\text{k}}{{\text{a}}_{{{\text{SC}} - {\text{RIS}}}}} * {{\text{A}}_{{{\text{depot}} - {\text{RIS}}}}} + \left( {{{\text{Q}}_{\text{RIS}}}/{{\text{V}}_{{{\text{p}} - {\text{RIS}}}}}} \right) * {{\text{A}}_{{{\text{p}} - {\text{RIS}}}}} - \left( {{{\text{Q}}_{\text{RIS}}}/{{\text{V}}_{{{\text{c}} - {\text{RIS}}}}}} \right) * {{\text{A}}_{{{\text{c}} - {\text{RIS}}}}} - \left( {{\text{C}}{{\text{L}}_{\text{RIS}}}/{{\text{V}}_{{{\text{c}} - {\text{RIS}}}}}} \right) * {{\text{A}}_{{{\text{c}} - {\text{RIS}}}}} - ({\text{C}}{{\text{L}}_{\text{met}}}/{{\text{V}}_{{{\text{c}} - }}}} \hfill \\ {_{\text{RIS}}) * {{\text{A}}_{{{\text{c}} - {\text{RIS}}}}}} \hfill \\ } $$

$$ {\text{d}}\left( {{{\text{A}}_{{{\text{p}} - {\text{RIS}}}}}} \right)/{\text{dt}} = \left( {{{\text{Q}}_{\text{RIS}}}/{{\text{V}}_{{{\text{c}} - {\text{RIS}}}}}} \right) * {{\text{A}}_{{{\text{c}} - {\text{RIS}}}}} - \left( {{{\text{Q}}_{\text{RIS}}}/{{\text{V}}_{{{\text{p}} - {\text{RIS}}}}}} \right) * {{\text{A}}_{{{\text{p}} - {\text{RIS}}}}} $$

$$ {\text{d}}\left( {{{\text{A}}_{{{\text{depot}} - {\text{PALI}}}}}} \right)/{\text{dt}} = {\text{DOS}}{{\text{E}}_{{{\text{SC}} - {\text{PALI}}}}}/{\text{D}}{{\text{R}}_{\text{PALI}}}\left( {{\text{t}} < {\text{D}}{{\text{R}}_{\text{SC--PALI}}}} \right) - {\text{k}}{{\text{a}}_{{{\text{SC}} - {\text{PALI}}}}} * {{\text{A}}_{{{\text{depot}} - {\text{PALI}}}}} $$

$$ \matrix{{*{20}{c}} {{\text{d}}\left( {{{\text{A}}_{{{\text{c}} - {\text{PALI}}}}}} \right)/{\text{dt}} = {\text{k}}{{\text{a}}_{{{\text{SC}} - {\text{PALI}}}}} * {{\text{A}}_{{{\text{depot}} - {\text{PALI}}}}} + \left( {{{\text{Q}}_{\text{PALI}}}/{{\text{V}}_{{{\text{p}} - {\text{PALI}}}}}} \right) * {{\text{A}}_{{{\text{p}} - {\text{PALI}}}}} - \left( {{{\text{Q}}_{\text{PALI}}}/{{\text{V}}_{{{\text{c}} - {\text{PALI}}}}}} \right) * {{\text{A}}_{{{\text{c}} - {\text{PALI}}}}} - \left( {{\text{C}}{{\text{L}}_{\text{PALI}}}/{{\text{V}}_{{{\text{c}} - {\text{PALI}}}}}} \right) * {{\text{A}}_{{{\text{c}} - {\text{PALI}}}}} + } \hfill \\ {\left( {{\text{C}}{{\text{L}}_{\text{met}}}/{{\text{V}}_{{{\text{c}} - {\text{RIS}}}}}} \right) * {{\text{A}}_{{{\text{c}} - {\text{RIS}}}}}} \hfill \\ } $$

$$ {\text{d}}\left( {{{\text{A}}_{{{\text{p}} - {\text{PALI}}}}}} \right)/{\text{dt}} = \left( {{\text{Q}_{\text{PALI}}}/{{\text{V}}_{{{\text{c}} - {\text{PALI}}}}}} \right) * {{\text{A}}_{{{\text{c}} - {\text{PALI}}}}} - \left( {{\text{Q} {\text{PALI}}}/{{\text{V}}_{{{\text{p}} - {\text{PALI}}}}}} \right) * {{\text{A}}_{{{\text{p}} - {\text{PALI}}}}} $$

Differential equations used to describe brain kinetics and receptor binding:

$$ \matrix{{*{20}{c}} {{\text{d}}\left( {{{\text{A}}_{{{\text{bv}} - {\text{RIS}}}}}} \right)/{\text{dt}} = \left( {{\text{C}}{{\text{L}}_{\text{bv}}}/{{\text{V}}_{\text{c}}}} \right) * {{\text{A}}_{{{\text{c}} - {\text{RIS}}}}} - \left( {{\text{C}}{{\text{L}}_{\text{bv}}}/{{\text{V}}_{\text{bv}}}} \right) * {{\text{A}}_{{{\text{bv}} - {\text{RIS}}}}} - \left( {{\text{C}}{{\text{L}}_{{{\text{bev}} - {\text{RIS}}}}}/{{\text{V}}_{\text{bv}}}} \right) * {\text{f}}{{\text{u}}_{{{\text{plasma}} - {\text{RIS}}}}} * {{\text{A}}_{{{\text{bv}} - {\text{RIS}}}}} + \left( {{\text{C}}{{\text{L}}_{{{\text{bev}} - {\text{RIS}}}}}/{{\text{V}}_{\text{bev}}}} \right) * {\text{f}}{{\text{u}}_{{{\text{brain}} - }}}} \hfill \\ {_{\text{RIS}} * {{\text{A}}_{{{\text{bev}} - {\text{RIS}}}}} + \left( {{\text{C}}{{\text{L}}_{{{\text{efflux}} - {\text{RIS}}}}}/{{\text{V}}_{\text{bev}}}} \right) * {\text{f}}{{\text{u}}_{{{\text{brain}} - {\text{RIS}}}}} * {{\text{A}}_{{{\text{bevF}} - {\text{RIS}}}}}} \hfill \\ } $$

$$ \matrix{{*{20}{c}} {{\text{d}}\left( {{{\text{A}}_{{{\text{bev}} - {\text{RIS}}}}}} \right)/{\text{dt}} = \left( {{\text{C}}{{\text{L}}_{{{\text{bev}} - {\text{RIS}}}}}/{{\text{V}}_{\text{bv}}}} \right) * {\text{f}}{{\text{u}}_{{{\text{plasma}} - {\text{RIS}}}}} * {{\text{A}}_{{{\text{bv}} - {\text{RIS}}}}} - \left( {{\text{C}}{{\text{L}}_{{{\text{bev}} - {\text{RIS}}}}}/{{\text{V}}_{\text{bev}}}} \right) * {\text{f}}{{\text{u}}_{{{\text{brain}} - {\text{RIS}}}}} * {{\text{A}}_{{{\text{bev}} - {\text{RIS}}}}} - \left( {{\text{C}}{{\text{L}}_{{{\text{efflux}} - {\text{RIS}}}}}/{{\text{V}}_{\text{bev}}}} \right) * {\text{f}}{{\text{u}}_{{{\text{brain}} - }}}} \hfill \\ {_{\text{RIS}} * {{\text{A}}_{{{\text{bev}} - {\text{RIS}}}}} - \left( {{\text{C}}{{\text{L}}_{\text{str}}}/{{\text{V}}_{\text{bev}}}} \right) * {\text{f}}{{\text{u}}_{{{\text{brain}} - {\text{RIS}}}}} * {{\text{A}}_{{{\text{bev}} - {\text{RIS}}}}} + \left( {{\text{C}}{{\text{L}}_{\text{str}}}/{{\text{V}}_{\text{str}}}} \right) * {\text{f}}{{\text{u}}_{{{\text{brain}} - {\text{RIS}}}}} * {{\text{A}}_{{{\text{strF}} - {\text{RIS}}}}} - \left( {{\text{C}}{{\text{L}}_{\text{cor}}}/{{\text{V}}_{\text{bev}}}} \right) * {\text{f}}{{\text{u}}_{{{\text{brain}} - {\text{RIS}}}}} * {{\text{A}}_{{{\text{bev}} - }}}} \hfill \\ {_{\text{RIS}} + \left( {{\text{C}}{{\text{L}}_{\text{cor}}}/{{\text{V}}_{\text{cor}}}} \right) * {\text{f}}{{\text{u}}_{{{\text{brain}} - {\text{RIS}}}}} * {{\text{A}}_{{{\text{corF}} - {\text{RIS}}}}}} \hfill \\ } $$

$$ \matrix{{*{20}{c}} {{\text{d}}\left( {{{\text{A}}_{{{\text{strF}} - {\text{RIS}}}}}} \right)/{\text{dt}} = \left( {{\text{C}}{{\text{L}}_{\text{str}}}/{{\text{V}}_{\text{bev}}}} \right) * {\text{f}}{{\text{u}}_{{{\text{brain}} - {\text{RIS}}}}} * {{\text{A}}_{{{\text{bev}} - {\text{RIS}}}}} - \left( {{\text{C}}{{\text{L}}_{\text{str}}}/{{\text{V}}_{\text{str}}}} \right) * {\text{f}}{{\text{u}}_{{{\text{brain}} - {\text{RIS}}}}} * {{\text{A}}_{{{\text{strF}} - {\text{RIS}}}}} - {\text{ko}}{{\text{n}}_{\text{D2}}} * {\text{f}}{{\text{u}}_{{{\text{brain}} - {\text{RIS}}}}} * {{\text{A}}_{{{\text{strF}} - {\text{RIS}}}}} * ({{\text{B}}_{{{ \max }}}}_{{ - {\text{D2}}}} - } \hfill \\ {{\text{C}}{{\text{B}}_{{{\text{D2}} - {\text{RIS}}}}} - {\text{C}}{{\text{B}}_{{{\text{D2}} - {\text{PALI}}}}}) + {\text{kof}}{{\text{f}}_{\text{D2}}} * {{\text{A}}_{{{\text{strB}} - {\text{RIS}}}}}} \hfill \\ } $$

$$ {\text{d}}\left( {{{\text{A}}_{{{\text{strB}} - {\text{RIS}}}}}} \right)/{\text{dt}} = {\text{ko}}{{\text{n}}_{\text{D2}}} * {\text{f}}{{\text{u}}_{{{\text{brain}} - {\text{RIS}}}}} * {{\text{A}}_{{{\text{strF}} - {\text{RIS}}}}} * ({{\text{B}}_{{{ \max }}}}_{{ - {\text{D2}}}} - {\text{C}}{{\text{B}}_{{{\text{D2}} - {\text{RIS}}}}} - {\text{C}}{{\text{B}}_{{{\text{D2}} - {\text{PALI}}}}}) - {\text{kof}}{{\text{f}}_{\text{D2}}} * {{\text{A}}_{{{\text{strB}} - {\text{RIS}}}}} $$

$$ \matrix{{*{20}{c}} {{\text{d}}\left( {{{\text{A}}_{{{\text{corF}} - {\text{RIS}}}}}} \right)/{\text{dt}} = \left( {{\text{C}}{{\text{L}}_{\text{cor}}}/{{\text{V}}_{\text{bev}}}} \right) * {\text{f}}{{\text{u}}_{{{\text{brain}} - {\text{RIS}}}}} * {{\text{A}}_{{{\text{bev}} - {\text{RIS}}}}} - \left( {{\text{C}}{{\text{L}}_{\text{cor}}}/{{\text{V}}_{\text{cor}}}} \right) * {\text{f}}{{\text{u}}_{{{\text{brain}} - {\text{RIS}}}}} * {{\text{A}}_{{{\text{corF}} - {\text{RIS}}}}} - {\text{ko}}{{\text{n}}_{{5}}}_{{ - {\text{HT2A}}}} * {\text{f}}{{\text{u}}_{{{\text{brain}} - {\text{RIS}}}}} * {{\text{A}}_{{{\text{corF}} - }}}} \hfill \\ {_{\text{RIS}} * ({{\text{B}}_{{{ \max }}}}_{{ - {5} - {\text{HT2A}}}} - {\text{C}}{{\text{B}}_{{{5} - {\text{HT2A}}--{\text{RIS}}}}} - {\text{C}}{{\text{B}}_{{{5} - {\text{HT2A}} - {\text{PALI}}}}}) + {\text{kof}}{{\text{f}}_{{5}}}_{{ - {\text{HT2A}}}} * {{\text{A}}_{{{\text{corB}} - {\text{RIS}}}}}} \hfill \\ } $$

$$ {\text{d}}\left( {{{\text{A}}_{{{\text{corB}} - {\text{RIS}}}}}} \right)/{\text{dt}} = {\text{ko}}{{\text{n}}_{{5}}}_{{ - {\text{HT2A}}}} * {\text{f}}{{\text{u}}_{{{\text{brain}} - {\text{RIS}}}}} * {{\text{A}}_{{{\text{corF}} - {\text{RIS}}}}} * ({{\text{B}}_{{{ \max }}}}_{{ - {5} - {\text{HT2A}}}} - {\text{C}}{{\text{B}}_{{{5} - {\text{HT2A}} - {\text{RIS}}}}} - {\text{C}}{{\text{B}}_{{{5} - {\text{HT2A}} - {\text{PALI}}}}}) - {\text{kof}}{{\text{f}}_{{5}}}_{{ - {\text{HT2A}}}} * {{\text{A}}_{{{\text{corB}} - {\text{RIS}}}}} $$

$$ \matrix{{*{20}{c}} {{\text{d}}\left( {{{\text{A}}_{{{\text{bv}} - {\text{PALI}}}}}} \right)/{\text{dt}} = \left( {{\text{C}}{{\text{L}}_{\text{bv}}}/{{\text{V}}_{{{\text{c}} - {\text{PALI}}}}}} \right) * {{\text{A}}_{{{\text{c}} - {\text{PALI}}}}} - \left( {{\text{C}}{{\text{L}}_{\text{bv}}}/{{\text{V}}_{\text{bv}}}} \right) * {{\text{A}}_{{{\text{bv}} - {\text{PALI}}}}} - \left( {{\text{C}}{{\text{L}}_{{{\text{bev}} - {\text{PALI}}}}}/{{\text{V}}_{\text{bv}}}} \right) * {\text{f}}{{\text{u}}_{{{\text{plasma}} - {\text{PALI}}}}} * {{\text{A}}_{{{\text{bvv}} - }}}} \hfill \\ {_{\text{PALI}} + \left( {{\text{C}}{{\text{L}}_{\text{bev PALI}}}/{{\text{V}}_{\text{bev}}}} \right) * {\text{f}}{{\text{u}}_{{{\text{brain}} - {\text{PALI}}}}} * {{\text{A}}_{{{\text{bev}} - {\text{PALI}}}}} + \left( {{\text{C}}{{\text{L}}_{{{\text{efflux}} - {\text{PALI}}}}}/{{\text{V}}_{\text{bev}}}} \right) * {\text{f}}{{\text{u}}_{{{\text{brain}} - {\text{PALI}}}}} * {{\text{A}}_{{{\text{bev}} - {\text{PALI}}}}}} \hfill \\ } $$

$$ \begin{array}{*{20}l}  {{{\text{d}}{\left( {{\text{A}}_{{{\text{bev}} - {\text{PALI}}}} } \right)}/{\text{dt}} = {\left( {{\text{CL}}_{{{\text{bev}} - {\text{PALI}}}} /{\text{V}}_{{{\text{bv}}}} } \right)} * {\text{fu}}_{{{\text{plasma}} - {\text{PALI}}}} * {\text{A}}_{{{\text{bv}} - {\text{PALI}}}} - {\left( {{\text{CL}}_{{{\text{bev}} - {\text{PALI}}}} /{\text{V}}_{{{\text{bev}}}} } \right)} * {\text{fu}}_{{{\text{brain}} - {\text{PALI}}}} * {\text{A}}_{{{\text{bev}} - {\text{PALI}}}} - ({\text{CL}}_{{{\text{efflux}} - {\text{PALI}}}} } \hfill} \\  {{/{\text{V}}_{{{\text{bev}}}} ) * {\text{fu}}_{{{\text{brain}} - {\text{PALI}}}} * {\text{A}}_{{{\text{bev}} - {\text{PALI}}}} - {\left( {{\text{CL}}_{{{\text{str}}}} /{\text{V}}_{{{\text{bev}}}} } \right)} * {\text{fu}}_{{{\text{brain}} - {\text{PALI}}}} * {\text{A}}_{{{\text{bev}} - {\text{PALI}}}} + {\left( {{\text{CL}}_{{{\text{str}}}} /{\text{V}}_{{{\text{str}}}} } \right)} * {\text{fu}}_{{{\text{brain}} - {\text{PALI}}}} * {\text{A}}_{{{\text{strF}} - {\text{PALI}}}} - } \hfill} \\  {{{\left( {{\text{CL}}_{{{\text{cor}}}} /{\text{V}}_{{{\text{bev}}}} } \right)} * {\text{fu}}_{{{\text{brain}} - {\text{PALI}}}} * {\text{A}}_{{{\text{bev}} - {\text{PALI}}}} + {\left( {{\text{CL}}_{{{\text{cor}}}} /{\text{V}}_{{{\text{cor}}}} } \right)} * {\text{fu}}_{{{\text{brain}} - {\text{PALI}}}} * {\text{A}}_{{{\text{corF}} - {\text{PALI}}}} } \hfill} \\ \end{array} $$

$$ {\text{d}}{\left( {{\text{A}}_{{{\text{strF}} - {\text{PALI}}}} } \right)}/{\text{dt}} = {\left( {{\text{CL}}_{{{\text{str}}}} /{\text{V}}_{{{\text{bev}}}} } \right)}*{\text{fu}}_{{{\text{brain}} - {\text{PALI}}}} *{\text{A}}_{{{\text{bev}} - {\text{PALI}}}}  - {\left( {{\text{CL}}_{{{\text{str}}}} /{\text{V}}_{{{\text{str}}}} } \right)}*{\text{fu}}_{{{\text{brain}} - {\text{PALI}}}} *{\text{A}}_{{{\text{strF}} - {\text{PALI}}}}  - {\text{kon}}_{{{\text{D}}2}} *{\text{fu}}_{{{\text{brain}} - {\text{PALI}}}} *{\text{A}}_{{{\text{strF}} - {\text{PALI}}}} *{\left( {{\text{B}}_{{\max  - {\text{D}}2}}  - {\text{CB}}_{{{\text{D}}2 - {\text{RIS}}}}  - {\text{CB}}_{{{\text{D}}2 - {\text{PALI}}}} } \right)} + {\text{koff}}_{{{\text{D}}2}} *{\text{A}}_{{{\text{strB}} - {\text{PALI}}}} $$

$$ {\text{d}}\left( {{{\text{A}}_{{{\text{strB}} - {\text{PALI}}}}}} \right)/{\text{dt}} = {\text{ko}}{{\text{n}}_{\text{D2}}} * {\text{f}}{{\text{u}}_{{{\text{brain}} - {\text{PALI}}}}} * {{\text{A}}_{{{\text{strF}} - {\text{PALI}}}}} * ({{\text{B}}_{{{ \max } - }}}_{\text{D2}} - {\text{C}}{{\text{B}}_{{{\text{D2}} - {\text{RIS}}}}} - {\text{C}}{{\text{B}}_{{{\text{D2}} - {\text{PALI}}}}}) - {\text{kof}}{{\text{f}}_{\text{D2}}} * {{\text{A}}_{{{\text{strB}} - {\text{PALI}}}}} $$

$$ \matrix{{*{20}{c}} {{\text{d}}\left( {{{\text{A}}_{{{\text{corF}} - {\text{PALI}}}}}} \right)/{\text{dt}} = \left( {{\text{C}}{{\text{L}}_{\text{cor}}}/{{\text{V}}_{\text{bev}}}} \right) * {\text{f}}{{\text{u}}_{{{\text{brain}} - {\text{PALI}}}}} * {{\text{A}}_{{{\text{bev}} - {\text{PALI}}}}} - \left( {{\text{C}}{{\text{L}}_{\text{cor}}}/{{\text{V}}_{\text{cor}}}} \right) * {\text{f}}{{\text{u}}_{{{\text{brain}} - {\text{PALI}}}}} * {{\text{A}}_{{{\text{corF}} - {\text{PALI}}}}} - {\text{ko}}{{\text{n}}_{{5}}}_{{ - {\text{HT2A}}}} * {\text{f}}{{\text{u}}_{{{\text{brain}} - }}}} \hfill \\ {_{\text{PALI}} * {{\text{A}}_{{{\text{corF}} - {\text{PALI}}}}} * ({{\text{B}}_{{{ \max }}}}_{{ - {5} - {\text{HT2A}}}} - {\text{C}}{{\text{B}}_{{{5} - {\text{HT2A}}--{\text{RIS}}}}} - {\text{C}}{{\text{B}}_{{{5} - {\text{HT2A}} - {\text{PALI}}}}}) + {\text{kof}}{{\text{f}}_{{5}}}_{{ - {\text{HT2A}}}} * {{\text{A}}_{{{\text{corB}} - {\text{PALI}}}}}} \hfill \\ } $$

$$ \matrix{{*{20}{c}} {{\text{d}}\left( {{{\text{A}}_{{{\text{corB}} - {\text{PALI}}}}}} \right)/{\text{dt}} = {\text{ko}}{{\text{n}}_{{5}}}_{{ - {\text{HT2A}}}} * {\text{f}}{{\text{u}}_{{{\text{brain}} - {\text{PALI}}}}} * {{\text{A}}_{{{\text{corF}} - {\text{PALI}}}}} * ({{\text{B}}_{{{ \max }}}}_{{ - {5} - {\text{HT2A}}}} - {\text{C}}{{\text{B}}_{{{5} - {\text{HT2A }}--{\text{RIS}}}}} - {\text{C}}{{\text{B}}_{{{5} - {\text{HT2A}} - {\text{PALI}}}}}) - {\text{kof}}{{\text{f}}_{{5}}}_{ - }} \hfill \\ {_{\text{HT2A}} * {{\text{A}}_{{{\text{corB}} - {\text{PALI}}}}}} \hfill \\ } $$

Where subscripts RIS and PALI denote parameters referring to risperidone and paliperidone, respectively. Subscripts depot, p, c, bv, bev, strF, strB, corF, corB represent volumes (V) and amounts (A) in depot (only for SC dosing), central, peripheral, brain vascular, brain extravascular, striatum free, striatum bound, frontal cortex free, frontal cortex bound compartments. DR_SC_ represents duration of zero-order absorption process for SC dosing. ka_SC_ is the absorption rate constant for SC dosing. CL, Q, CL_bv_, CL_str_, CL_cor_ represent clearance in the central, peripheral, brain vascular, striatum free, cortex free compartments, respectively. CL_met_ represent metabolic conversion of RIS to PALI. CL_bev_ and CL_efflux_ represent passive diffusion and active efflux through the BBB. B_max_ is the receptor density. CB are concentrations bound to receptor in nM. They are calculated as follows:

$$ {\text{C}}{{\text{B}}_{{{\text{D2}} - {\text{RIS}}}}} = {1}000 * {{\text{A}}_{{{\text{strB}} - {\text{RIS}}}}}/{{\text{V}}_{\text{str}}}/{\text{M}}{{\text{W}}_{\text{RIS}}} $$

$$ {\text{C}}{{\text{B}}_{{{\text{D2}} - {\text{PALI}}}}} = {1}000 * {{\text{A}}_{{{\text{strB}} - {\text{PALI}}}}}/{{\text{V}}_{\text{str}}}/{\text{M}}{{\text{W}}_{\text{PALI}}} $$

$$ {\text{C}}{{\text{B}}_{{{5} - {\text{HT2A}} - {\text{RIS}}}}} = {1}000 * {{\text{A}}_{{{\text{corB}} - {\text{RIS}}}}}/{{\text{V}}_{\text{cor}}}/{\text{M}}{{\text{W}}_{\text{RIS}}} $$

$$ {\text{C}}{{\text{B}}_{{{5} - {\text{HT2A}} - {\text{PALI}}}}} = {1}000 * {{\text{A}}_{{{\text{corB}} - {\text{PALI}}}}}/{{\text{V}}_{\text{cor}}}/{\text{M}}{{\text{W}}_{\text{PALI}}} $$

Where MW is a molecular weight.

RO is calculated as $({\text{C}}{{\text{B}}_{\text{RIS}}} + {\text{C}}{{\text{B}}_{\text{PALI}}})/{{\text{B}}_{{{ \max }}}}$.

## ABBREVIATIONS

BBB: blood-brain barrier
B_max_: receptor density
CL: systemic clearance
CL_bev_: passive brain-extravascular clearance
CL_bv_: brain-vascular clearance
CL_cor_: brain-cortex clearance
CL_efflux_: active efflux clearance
CL_met_: metabolic clearance of RIS to PALI
CL_PALI_: systemic clearance of PALI
CL_RIS_: systemic clearance of RIS by other routes than metabolism to PALI
CL_str_: brain-striatum clearance
CWRES: conditional weighted residuals
F_IP_: bioavailability for intraperitoneal route of administration
FOCE: first order conditional estimation method
Fr_FPM_: fraction of the absorbed RIS IP dose going directly to PALI central compartment
F_SC_: bioavailability for subcutaneous route of administration
fu_brain_: unbound fraction in brain
fu_plasma_: unbound fraction in plasma
GOF: goodness-of-fit plots
IIV: inter-individual variability
IP: intraperitoneal
IV: intravenous
Kd: receptor dissociation constant
koff: receptor dissociation rate constant
kon: receptor association rate constant
nM: nanomoles/litre
OFV: objective function value
PALI: paliperidone
P-gp: P-glycoprotein
PK-PD: pharmacokinetic and pharmacodynamic
Q: intercompartmental flow between central and peripheral compartment
RIS: risperidone
RO: receptor occupancy
RSE: relative Standard Error
SC: subcutaneous
V_bev_: volume of brain-extravascular compartment
V_bv_: volume of brain-vascular compartment
V_c_: volume of the central compartment
V_cor_: volume of frontal cortex compartment
V_p_: volume of the peripheral compartment
V_str_: volume of striatum compartment
